# Glycosphingolipids and Infection. Potential New Therapeutic Avenues

**DOI:** 10.3389/fcell.2019.00324

**Published:** 2019-12-06

**Authors:** Johannes M. F. G. Aerts, M. Artola, M. van Eijk, M. J. Ferraz, R. G. Boot

**Affiliations:** Leiden Institute of Chemistry, Leiden University, Leiden, Netherlands

**Keywords:** glycosphingolipid, infection, glucosylceramide, lysosome, glycosidase, glycosyltransferase

## Abstract

Glycosphingolipids (GSLs), the main topic of this review, are a subclass of sphingolipids. With their glycans exposed to the extracellular space, glycosphingolipids are ubiquitous components of the plasma membrane of cells. GSLs are implicated in a variety of biological processes including specific infections. Several pathogens use GSLs at the surface of host cells as binding receptors. In addition, lipid-rafts in the plasma membrane of host cells may act as platform for signaling the presence of pathogens. Relatively common in man are inherited deficiencies in lysosomal glycosidases involved in the turnover of GSLs. The associated storage disorders (glycosphingolipidoses) show lysosomal accumulation of substrate(s) of the deficient enzyme. In recent years compounds have been identified that allow modulation of GSLs levels in cells. Some of these agents are well tolerated and already used to treat lysosomal glycosphingolipidoses. This review summarizes present knowledge on the role of GSLs in infection and subsequent immune response. It concludes with the thought to apply glycosphingolipid-lowering agents to prevent and/or combat infections.

## Introduction to Glycosphingolipids

Glycosphingolipids (GSLs) were discovered by the German chemist Johannes Thudichum while investigating the composition of the human brain in his London laboratory in the late 19th century (Thudichum, 1884). Thudichum meticulously identified the structure of the encountered novel class of lipids as consisting of a unique lipid moiety with attached sugar or phosphorylcholine groups. The hydrophobic moiety of the isolated brain lipids proved to contain as backbone a hitherto unknown D-erythro-Sphingosine, named after the mythical Sphinx for its “enigmatic properties to the enquirer.” The value of Thudichum’s findings was initially highly debated and did not meet recognition during his lifetime. Only 25 years after his death, Otto Rosenheim confirmed the accuracy of his publications which finally opened the present vast field of GSL research (King, 1956).

### Features of Glycosphingolipids

#### Structure of Glycosphingolipids

**FIGURE 1:**
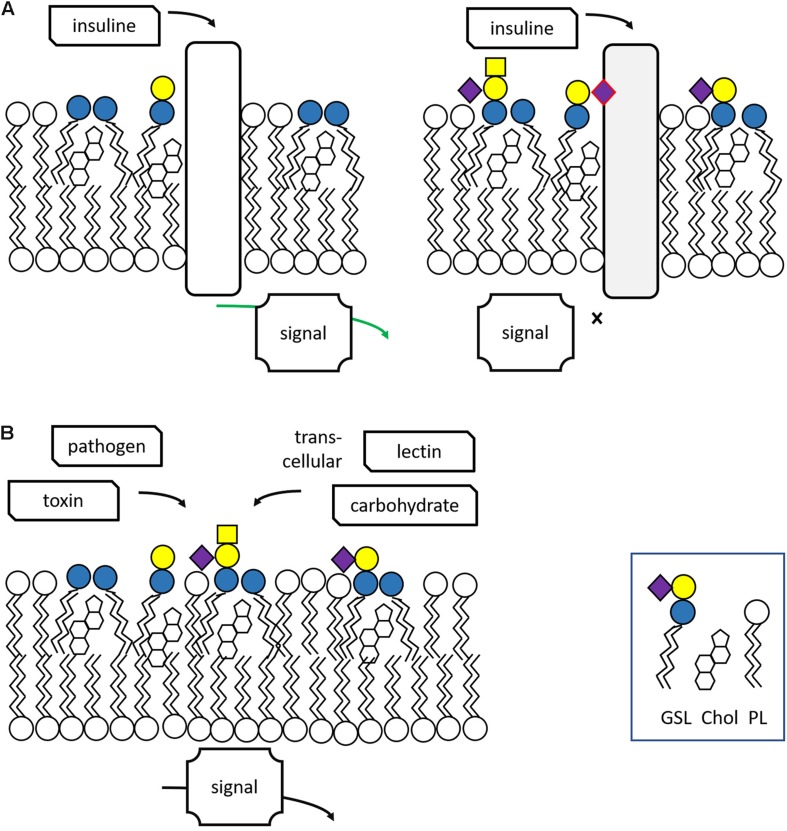
Structure and synthesis of glycosphingolipids. **(A)** Synthesis of complex glycosphingolipids (GSLs) from the simple building blocks L-serine, fatty acyl-CoA, and UDP-sugars. **(B)** General structure of glycosphingolipid: indicated are the major globo-, isoglobo-, ganglio-, lacto-, and neolacto-series core structures.

In vertebrates the major form of the sphingoid base of GSLs is d18:1 sphingosine, (2*S*,3*R*,4*E*)-2aminooctadec-4-ene-1,3-diol. *N*-acylation of sphingosine results in the lipid ceramide (Cer), the primary sphingolipid. The attachment of sugars to Cer yields GSLs (Figure 1A). Ubiquitous in brain is the simple GSL galactosylceramide (GalCer), the major lipid constituent of myelin insulating axons of neuronal cells and formed as extended plasma membrane of oligodendrocytes. Following Thudichum’s seminal findings, it became apparent that GSLs are not restricted to the brain but are common components of cells in various organisms. In the case of human GSLs, the first monosaccharide linked to Cer is either glucose or galactose. Additional sugars can be further attached to glucosylceramide (GlcCer) or GalCer, resulting in a plethora of lipids of which quantitatively the most abundant are the ganglio-, globo-, and neolacto-series of GSLs (Figure 1B). The structural diversity of GSLs and their nomenclature have been thoroughly reviewed (Wennekes et al., 2009; Merrill, 2011; Merrill and Sullards, 2017).

#### Synthesis of Glycosphingolipids

During their life in cells, GSL molecules traverse various subcellular compartments where specific modifications in their structure may occur (Wennekes et al., 2009; Gault et al., 2010; Merrill, 2011; Fabrias et al., 2012; D’Angelo et al., 2013; Tidhar and Futerman, 2013; Sandhoff and Sandhoff, 2018; Sandhoff et al., 2018). The synthesis starts at the endoplasmic reticulum (ER) where the enzyme serine palmitoyltransferase (SPT) generates keto-sphinganine from serine and palmitoyl-CoA (Figure 1A). This building block is next converted to sphinganine by 3-ketodihydrosphingosine reductase (KDSR). Subsequently, a series of ceramide synthases (CerS 1-6) form dihydroceramides with amide-linked fatty acyl moieties. Distinct dihydroceramides are generated since the various CerS enzymes have different acyl-CoA length preferences (Tidhar and Futerman, 2013). From these dihydroceramides the enzyme dihydroceramide desaturase-1 (DES1) forms Cer. The primary sphingolipid Cer is subject to further metabolism and its levels are tightly controlled in this manner, a necessity for cells since Cer promotes concentration-dependently apoptotic processes, and subsequent cell death (Zelnik et al., 2019). Cer may be converted into sphingomyelin (SM; phosphocholine-ceramide) or ceramide-1-phosphate (Cer1P). It may be also degraded by neutral ceramidases to fatty acid and sphingosine (Wennekes et al., 2009). A third metabolic route involves formation of structurally diverse GSLs through distinct pathways. In tissues like kidney and brain a large portion of newly formed Cer molecules enter the ER where conversion to GalCer occurs, catalyzed by the enzyme galactosylceramide synthase (CGT) using UDP-galactose as sugar donor (Sprong et al., 1998; van Meer et al., 2003). Sulfation of GalCer molecules may take place to generate sulfatide (sulfo-GalCer). More commonly, the Cer transfer protein (CERT) transports newly formed Cer to the cytosolic leaflet *cis-*Golgi membranes (Hanada et al., 2003, 2009). Here, the enzyme glucosylceramide synthase (GCS; encoded by the *UGCG* gene) may transform Cer to GlcCer using UDP-glucose as sugar donor (Ichikawa et al., 1996). Some of the GlcCer in the cytosolic membrane leaflet is metabolized back again to Cer by the enzyme GBA2, a cytosol-faced β-glucosidase that also shows transglucosylase activity (van Weely et al., 1993; Boot et al., 2007; Marques et al., 2016). However, most newly formed GlcCer enters the Golgi apparatus where it can be stepwise modified by glycosyltransferases (Wennekes et al., 2009; Merrill, 2011; Merrill and Sullards, 2017; Sandhoff and Sandhoff, 2018). The addition of further sugars to GlcCer yields various types of GSLs (Figure 1B). Increasing the vast diversity of GSLs is the sulfation of particular lipids. After being modified in the Golgi apparatus, GSLs end up in the outer leaflet of the plasma membrane. GSLs may partly leave cells through incorporation in HDL-lipoproteins (Van den Bergh and Tager, 1976).

Congenital human disorders of ganglioside biosynthesis are very rare. Mutations in ST3GAL5 (encoding GM3 synthase) cause severe congenital infantile seizures. Mutations in B4GALNT1 (encoding GM2/GD2 synthase) lead to hereditary spastic paraplegia accompanied by intellectual disability (Li and Schnaar, 2018).

#### Degradation

Glycosphingolipids are internalized via endocytosis and end up in multi-vesicular bodies in endosomes. Next, their fragmentation takes place in lysosomes (Cox and Cachón-González, 2012; Platt, 2014). Through endocytosis lysosomes acquire also exogenous GSLs. These are components of phagocytosed senescent cells and debris as well as endocytosed lipoproteins. In the acid lysosomes, GSLs are fragmented by a series of glycosidases in a stepwise manner (Ferraz et al., 2014; Breiden and Sandhoff, 2019). In this process, specific glycosidases remove terminal sugar moieties from GSLs, the reverse of the biosynthetic pathway. Many of the lysosomal glycosidases fragmenting GSLs are assisted in their activity by specific accessory proteins (GM2 activator protein and saposin A–D) (Ferraz et al., 2014; Breiden and Sandhoff, 2019). Cer, the lipid product of lysosomal GSL degradation, is cleaved by the lysosomal acid ceramidase into sphingosine and fatty acid. The degradation products (sugars, fatty acids, and sphingosine) are exported to the cytosol. The exported sphingosine may be next re-used in the salvage pathway that generates again Cer molecules for the synthesis of SM or GSLs. Alternatively, sphingosine is transformed by sphingosine kinases (SK1 and SK2) to sphingosine-1-phosphate (S1P). This may be subsequently degraded by S1P lyase into phosphatidylethanolamine and 2-trans-hexadecenal (Pyne et al., 2016).

### Functions of Glycosphingolipids

#### Lipid Raft Signaling Platforms

**FIGURE 2:**
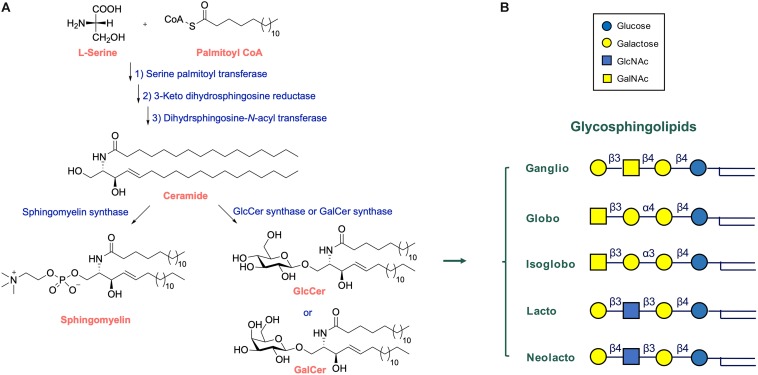
Lipid rafts and other functions of glycosphingolipids. **(A)** Glycosphingolipids are essential components of lipid rafts where signaling events occur in response to extracellular triggers. Excessive GSLs (GM3) may interfere with signaling. **(B)** GSLs may interact with toxins, bacteria and trans-cellular lectins and carbohydrates. Adapted from Feingold and Elias (2014).

Glycosphingolipids reside primarily in the cellular plasma membrane with their sugar moieties exposed to the exterior. At the cell surface, GSLs have multiple functions. Through interactions among GSL molecules and cholesterol molecules via hydrogen bonds and van der Waal’s forces semi-ordered domains spontaneously form in the plasma membrane. In these lipid rafts specific proteins involved in signaling events locate (Mukherjee and Maxfield, 2004; Lingwood and Simons, 2010; Sonnino and Prinetti, 2013; Figure 2A). It has become clear that GSLs in lipid rafts may regulate the activity of some of these signaling receptors. A particularly well studied example of the impact of gangliosides on receptor signaling concerns the epidermal growth factor receptor (EGFR). Well-established is the inhibitory effect of GM3 on the receptor’s kinase domain activation, a phenomenon abolished by conversion of GM3 to lactosylceramide (LacCer) or the K642G amino acid substitution in the EGFR (Coskun et al., 2011). Thus, GM3 modulates the allosteric structural transition from inactive to signaling EGFR dimer. Another example forms the insulin receptor whose activity is influenced by local gangliosides (Kabayama et al., 2007; Langeveld and Aerts, 2009). Obese mice genetically unable to synthetize the ganglioside GM3 show better glucose tolerance and insulin sensitivity than control obese animals (Tagami et al., 2002; Yamashita et al., 2003). Pharmacological reduction of GSLs, including that of gangliosides, improves markedly insulin sensitivity and glucose homeostasis in obese rodents (Aerts et al., 2007; Zhao et al., 2009). Of note, patients with Gaucher disease (GD) (see section “Lysosomal Glycosphingolipid Storage Disorders and Therapy” for a detailed description of this inherited disorder) show elevated levels of the gangliosides GM3 in cells and tissue and in parallel reduced insulin sensitivity (Ghauharali-van der Vlugt et al., 2008; Langeveld et al., 2008).

A similar modulatory role for gangliosides has also been noted for other receptors such as the T-cell receptor amongst others (Inokuchi et al., 2018). Recently gangliosides were found to also impact on the activity of the membrane embedded protein NPC1L1, critically involved in intestinal cholesterol absorption (Nihei et al., 2018). Another intriguing finding is that the ganglioside GM1 prevents oligomerization of b-amyloid oligomers, whilst SM promotes this (Amaro et al., 2016). This finding may proof to be relevant to design strategies to ameliorate Alzheimer’s disease (Amaro et al., 2016). LacCer-enriched lipid rafts have been identified in plasma and granular membranes of human neutrophils (see Nakayama et al., 2018 for a review). The first report on LacCer-raft mediated neutrophil function concerned superoxide generation (Iwabuchi and Nagaoka, 2002). It was demonstrated that the incubation of neutrophils with anti-LacCer antibody induced generation of superoxide. A key role for activation of Lyn in the process was identified (Iwabuchi and Nagaoka, 2002).

Glycosphingolipids have been found to also interact other cells, either via protein-carbohydrate or carbohydrate-carbohydrate interactions (Figure 2B). The proteins involved in such interactions are three major classes of lectins: selectins binding sialylated and fucosylated glycans; siglecs binding galectins and sialylated glycans; and galectins binding glycans containing terminal galactose (Schnaar, 2004).

In land animals, GSLs fulfill a special function in the outermost skin, the stratum corneum. Here, extraordinary GlcCer molecules with very-long-chain fatty acid (C30–32) and an ω-hydroxyl group esterified to another fatty acid are obligate precursors to Cer that are locally required to build the desired protective and permeability layer (Feingold and Elias, 2014; Van Smeden and Bouwstra, 2016; Wertz, 2018). Disturbance in skin GlcCer and Cer are associated with severe, even fatal, dysfunction of the skin (Van Smeden et al., 2017). The presence of specific gangliosides in neurons has multiple functions and proves to be essential for optimal interplay with the insulating myelin (Lopez and Báez, 2018). In particular, lack of specific gangliosides in axons of neurons leads to disturbed interaction with myelin-associated glycoprotein (MAG) in the innermost membrane of myelin. This impairment is thought to underly the spastic paraplegia during neuronal deficiency of specific gangliosides (Schnaar and Lopez, 2009).

Exposed glycans of GSLs on epithelial cells contribute to the protective properties of the glycocalyx of internal body linings. A similar type of protective function of GSLs is envisioned for lysosomes inside cells. Beside the outer leaflet plasma membrane, the inner leaflet of the lysosomal membrane is rich in GSLs. This membrane also contains integral membrane proteins that are decorated with N-linked glycans. By the combined presence of GSLs and membrane glycoproteins the lysosomal membrane is thought to be protected by a sugar barrier against self-degradation by the proteases and lipases present in the lumen of the compartment (Schwake et al., 2013).

Specific GSLs at the surface of cells also undergo specific interactions with the outside world. For example, some GSLs contain the glycan-based ABO antigens, crucial in self-recognition and of importance in transfusion medicine (Kościelak, 2012). E-selectin mediated binding of tissue invading leukocytes to endothelial cells is known to be dependent on specific GSLs (Nimrichter et al., 2008).

### Glycosphingolipids and Infection

#### Interaction With Pathogens and Toxins

**FIGURE 3:**
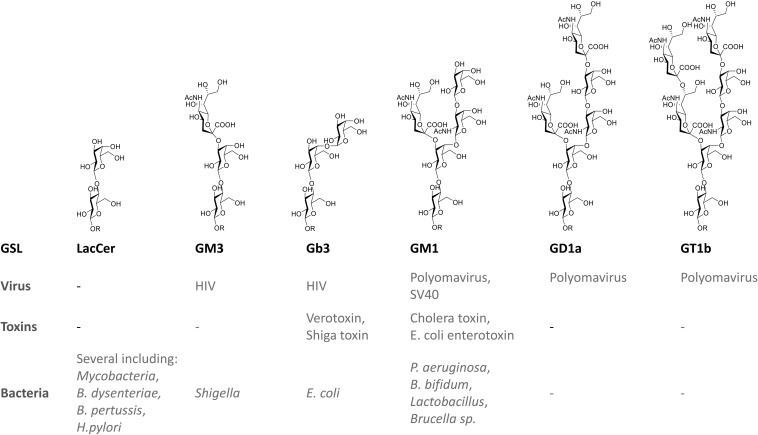
Examples of direct interactions of glycosphingolipids with pathogens and toxins.

Many viruses, bacteria, and bacterial toxins bind to carbohydrates of GSLs on host cell surfaces (Figure 3). Recommended reviews of the topic are Nakayama et al. (2018) and Hanada (2005).

##### Viruses

Some viruses are known to exploit GSLs in their life cycle in hosts (Lingwood, 1996). One well studied example is the human immunodeficiency virus (HIV) (Lingwood and Branch, 2011). HIV virions enter their host cells by the binding of the viral envelope gp120 to the primary receptor CD4, an essential interaction for viral fusion and entry. G protein-coupled α- and β-chemokine receptors are required as “co-receptors” together with CD4 for HIV-1 infection. GSLs display a complex interaction with HIV gp120, with reports suggesting functions as alternate entry receptors, facilitators for HIV infection, as well as natural resistance factors for HIV infection. Several GSLs (Gb3, GM3, GalCer, sulfatide) appear to bind the HIV adhesin gp120 (Lund et al., 2009; Lingwood and Branch, 2011). Upon binding, GSLs such as GalCer and GM3 facilitate subsequent interaction of the virus with its chemokine co-receptor on the cell surface (Lingwood and Branch, 2011). In contrast, Gb3 may successfully compete for co-receptor binding, and prevent fusion and viral entry (Lund et al., 2009; Lingwood and Branch, 2011). *Polyomavirus* invades human erythrocytes via the gangliosides GD1a and GT1b (Schwake et al., 2013). GM1 has also been shown to act as receptors for simian virus 40 (SV40) and polyoma virus (Tsai et al., 2003).

##### Toxins

Protein toxins show an AB structure, with a catalytic A domain and a B domain encoding host receptor recognition (Zuverink and Barbieri, 2018). Gb3, (a.k.a. CD77 or P(k) blood group antigen) is known to bind to Shiga toxin and the closely related *Escherichia coli* (*E. coli*) derived verotoxin B subunit (van Heyningen, 1974). The globoside thus is mediating verotoxin induced hemolytic uremic syndrome (HUS) (Lingwood, 1996). The ganglioside GM1 serves as the primary receptor for cholera toxin and the highly homologous *E. coli* heat-labile enterotoxin (Hirst et al., 2002). *Clostridium tetani* neurotoxin and *Clostridium botulinum* neurotoxin type A and B use several gangliosides as receptors (Kitamura et al., 1999). The ganglioside GM2 acts as a receptor for delta-toxin of *Clostridium perfringens* (Jolivet-Reynaud et al., 1989). Cholera toxin B subunit (CTB) binds to GM1 enriched in lipid rafts (Cuatrecasas, 1973a, b). GM1 on epithelial cells also binds *E. coli* enterotoxin (Hyun and Kimmich, 1984; Masserini et al., 1992; Kuziemko et al., 1996). The gangliosides present in human milk are thought to compete the binding of *Vibrio cholerae* and *E. coli* enterotoxins in the intestine and thus offer protection (Otnaess et al., 1983; Newburg and Chaturvedi, 1992).

##### Bacteria

The ganglioside asialo-GM1 (GA1) at the surface of epithelial cells binds *Bifidobacterium bifidum*, *Pseudomonas aeruginosa*, and *Lactobacillus* (de Bentzmann et al., 1996; Mukai et al., 2004). The ganglioside GM1 has been implicated in infections with *Brucella* species (Naroeni and Porte, 2002; Martín-Martín et al., 2010). Fimbriated *E. coli* bind to the globosides Gb3 and Gb4 (Leffler and Svanborg-Edén, 1981). Virulent strains of *Bordetella pertussis*, a human respiratory pathogen, bind with high affinity to sulfatide (Brennan et al., 1991). *Mycoplasma pneumoniae* appears to exploit GSLs containing terminal Gal(3SO_4_)β1-residues (Krivan et al., 1989).

The neutral GSL LacCer at the surface of intestinal epithelial cells binds various microorganisms. These include *Candida albicans*, *B. pertussis*, *Mycobacterium tuberculosis*, *E. coli*, *Bacillus dysenteriae*, and *Propionibacterium freudenreichii* (Nakayama et al., 2018). Possibly milk-derived LacCer protects the host from invading pathogens. Interactions between the sugar moieties of gangliosides and the polysaccharide moieties of *Shigella* lipopolysaccharide were found to facilitate binding of bacteria to human CD4+ T cells (Belotserkovsky et al., 2018). There are indications that the adhesion of *Helicobacter pylori*, causing chronic active gastritis, peptic ulcer disease and gastric adenocarcinoma, depends on gangliosides in the human stomach. The gangliosides Neu5Acα3-neolactohexaosylceramide and Neu5Acα3-neolactooctaosylceramide mediate attachment of *H. pylori* SabA (sialic acid binding adhesin) there (Mahdavi et al., 2002; Benktander et al., 2018).

#### Immune System

The role of GSLs in immune cell functions receives increasing interest and has been recently reviewed (Zuidscherwoude et al., 2014; Zhang et al., 2019). Besides acting as entry point of pathogens and toxins, GSLs also impact on the response of the immune system to pathogens. As such, GSLs themselves can also transduce signals as revealed by the effect of their crosslinking by multivalent binders such as bacterial toxins, or alternatively IgM antibodies (Spiegel, 1989; Klokk et al., 2016). Influx of calcium ions upon cell surface crosslinking of GM1 seems to be largely mediated by L-type calcium channels (Carlson et al., 1994). As another example, in human neutrophils LacCer forms specific lipid rafts in the plasma membrane as well as granular membranes. These rafts have been shown to interact with β-glucan of *C. albicans* and lipoarabinomannan (LAM) of *Mycobacteria* (Sato et al., 2006; Nakayama et al., 2016). Such binding triggers signaling cascades involving Src family kinases. The responses to this are chemotaxis, phagocytosis, and phagolysosome formation. In neutrophils, *M. tuberculosis* smartly targets the LacCer-enriched lipid rafts in phagosomes to inhibit the maturation of phagosome to lytic phagolysosomes (Nakayama et al., 2018).

Other direct and indirect interactions of GSLs with immune cells affecting their activity have more recently come to light. For example, the C-type lectin receptor Mincle (macrophage inducible C-type lectin), contributes to innate immune responses by recognition of lipids stemming from foreign pathogens like glucose and trehalose mycolates and glycosyl diacylglycerols, but also lipids from damaged cells (Williams, 2017). Among the reported Mincle-interacting self antigens are sterols but also GlcCer (Nagata et al., 2017).

In the case of dendritic cells, glycolipid antigens are presented by MHC class I molecule (CD1d) of dendritic cells via T-cell receptor recognition to activate natural killer T (NKT) cells which control innate and adaptive immune responses (Kumar et al., 2017). The marine sponge GSL α-GalCer is identified as potent lipid antigen activating invariant NTK (iNKT) cells. These cells are also activated by the endogenous iGb3Cer (Galα1-3Galβ1-4GlcβCer) (Pei et al., 2012). More recently, excessive GlcCer has also been proposed to act as an iNKT cell activator (Nair et al., 2015). Of note, GlcCer synthase deficiency in mouse cells was already earlier reported to impair CD1d-dependent activation of iNKT cells, suggesting that GlcCer or its metabolites might be endogenous ligands for CD1d-restricted iNKT cells (Stanic et al., 2003).

In addition to modulating innate immunity, GSLs also appear to play critical roles in adaptive immunity. For example, gangliosides influence T cell receptors (TCRs) on CD-4 positive (CD4+) and CD-8 positive (CD8+) T cells, respectively (Nagafuku et al., 2012). Here it is thought that the precise ganglioside composition of lipid rafts in specific T cell populations is a prerequisite for their associated specific effector functions. This regulatory aspect of gangliosides in T cell biology seems highly relevant for allergic and autoimmune diseases and has been topic of excellent reviews (Inokuchi et al., 2018; Nakayama et al., 2018).

In some specific autoimmune neuropathies affecting the nervous system the autoimmune attack is due to antibodies reactive with gangliosides. Anti-ganglioside antibodies occur for example with Guillain–Barré syndrome. These antibodies may be induced by infections with pathogens containing glycan components that are structurally similar to gangliosides. The most important example of this is *Campylobacter jejuni* whose surface lipo-oligosaccharide mimics GD1a, GT1a, GM1, and other gangliosides (Goodfellow and Willison, 2018). Binding of autoantibodies on gangliosides activates locally complement and recruits macrophages, causing local impairment of nerve conduction in these patients.

#### Sphingomyelin and Infection

Sphingomyelin is the most abundant cellular sphingolipid. Like GSLs, SM is also implicated in infections and the immune system’s response to these (Wu et al., 2018; Li et al., 2019). For example, mice with deficiency of acid sphingomyelinase (ASMase; Sphingomyelin phosphodiesterase 1), the enzyme hydrolyzing SM to Cer and phosphorylcholine, are strongly susceptible to *Citrobacter rodentium*-driven colitis (Meiners et al., 2019). Mice overexpressing ASMase in T cells show increased T cell activation and reduced parasitemia in upon infection with *Plasmodium yoelii* (Hose et al., 2019). Two forms of ASMase are encoded by the *SMPD1* gene: a lysosomal form (L-ASMase) and a secretory form (S-ASMase). Although ASMase has an acid pH optimum for activity, the same enzyme, when secreted, also catalyzes the hydrolysis of SM in the circulation and on the plasma membrane (Smith and Schuchman, 2008; Schuchman, 2010). ASMase deficiency results in the accumulation of SM in lysosomes and causes the neuropathic (type A) and non-neuropathic (type B) variants of Niemann-Pick disease (Schuchman, 2010). Generation of Cer molecules on the cell surface by ASMase leads to formation of Cer-enriched domains, distinct from traditional lipid rafts, that act as platforms governing signaling events (Li et al., 2019). Cer-enriched platforms occur in cells upon diverse receptor or non-receptor stimuli, including CD95, FcγRII, CD40, platelet-activating factor receptor (PAF), viral infection, *P. aeruginosa*, *Neisseria gonorrhoeae*, *Staphylococcus aureus*, cisplatin, Cu^2+^, irradiation and UV-light (Li et al., 2019). The interaction of Cer-enriched platforms with CD95, the death receptor Fas, is the best understood. CD95 induces an increased ASMase activity on the cell surface, thus generating Cer-enriched platforms amplifying CD95 signaling (Gulbins and Grassmé, 2002; Grassmé et al., 2007).

The ASMase/Cer system has been implicated in infections with pathogens. *P. aeruginosa* is a gram-negative bacterium commonly affecting immune-compromised patients and patients with chronic wounds, sepsis, or chronic emphysema. Patients with cystic fibrosis (CF) have a particular risk for chronic *P. aeruginosa* infections. The infection of mammalian cells with different strains of *P. aeruginosa* induces the rapid activation of ASMase and translocation to the plasma membrane (Li et al., 2019). Generated Cer-rich rafts by ASMase mediates the internalization of *P. aeruginosa*, which is prevented by inhibitors of the ASMase or by ASMase-deficiency. Amitriptyline, a tricyclic antidepressant (TCA), is a functional ASMase inhibitor. Administration of amitriptyline to CF mice normalizes pulmonary Cer levels and abolishes pathological outcome, including susceptibility to infection (Becker et al., 2010). Amitriptyline might therefore offer in the future a novel medicine to treat CF patients.

ASMase appears critical in the regulation of host interactions with other bacteria as well, including *S. aureus*, *Mycobacteria*, *Listeria monocytogenes* and *Neisseria* species. *S. aureus*, is a commensal opportunistic bacterium that colonizes approximately 30% of human populations. It may cause life-threatening endocarditis, diseases, sepsis, toxic shock syndrome, and pneumonia (Li et al., 2019). *S. aureus* is the primary cause of sepsis and lethal lung edema. Mice treated with the ASMase inhibitor amitriptyline show reduced lung edema upon *S. aureus* exposure. The effect on sepsis of various ASMase inhibitors (imipramine, desipramine, and amitriptyline), is presently studied in animal models (Chung et al., 2018; Xia et al., 2019).

### Lysosomal Glycosphingolipid Storage Disorders and Therapy

**FIGURE 4:**
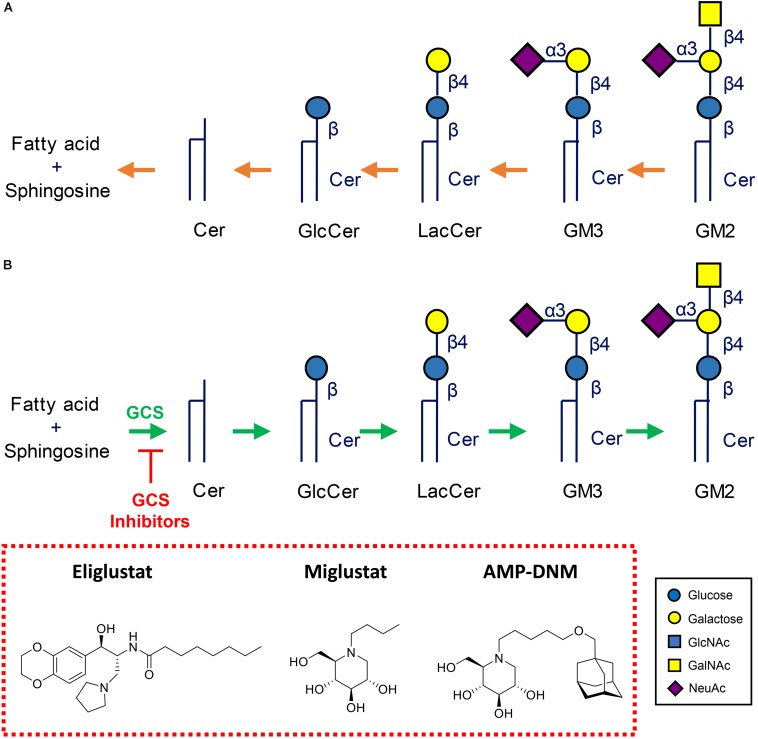
Metabolism of glycosphingolipids. **(A)** Lysosomal degradation by glycosidases assisted by activator proteins. Indicated are common lysosomal storage disorders stemming from inherited defects in lysosomal hydrolases. **(B)** Therapeutic reduction of glycosphingolipids by inhibition of glucosylceramide synthase (GCS). Shown are two clinically registered GCS inhibitors (Miglustat, *N*-butyl-deoxynojirimycin) and Eliglustat (*N*-[(1*R*,2*R*)-1-(2,3-Dihydro-1,4-benzodioxin-6-yl) -1-hydroxy-3-(1-pyrrolidinyl)-2-propanyl]octanamide), and AMP-DNM (*N*-(5-adamantane-1-yl-methoxy-pentyl)-deoxynojirimycin) commonly employed in research.

Inherited defects in lysosomal enzymes fragmenting GSLs lead to accumulation of the accompanying substrate in lysosomes. Several inherited lysosomal glycosphingolipid storage disorders (glycosphingolipidoses) occur in humans, see Figure 4 (Cox and Cachón-González, 2012; Ferraz et al., 2014; Platt, 2014; Breiden and Sandhoff, 2019).

#### Gaucher Disease

A prototype glycosphingolipidosis is GD, named after Ernest Gaucher who published the first case report (Beutler and Grabowski, 2001). GD is a recessively inherited disorder stemming from mutations in the *GBA* gene. This codes for an acid β-glucosidase, better known as glucocerebrosidase (GCase; EC. 3.2.1.45) (Brady et al., 1966; Beutler and Grabowski, 2001). The 497 amino acid glycoprotein cleaves GlcCer to Cer, the penultimate step in lysosomal breakdown of most GSLs. Prominent GlcCer accumulation characteristically occurs in tissue macrophages (Gaucher cells) of GD patients. The clinical presentation of GCase deficiency is very heterogeneous, from severe neonatal complications to a virtually asymptomatic course. Non-neuronopathic (type 1), acute neuronopathic (type 2), and sub-acute neuronopathic (type 3) GD phenotypes are discerned. A complete deficiency of GCase causes fatal skin pathology causing abnormal permeability properties (Beutler and Grabowski, 2001). It has recently been recognized that individuals with a mutant *GBA* allele are at increased risk, about 20-fold, to develop Parkinson disease (Siebert et al., 2014). Although some mutations in the *GBA* gene are associated with a benign GD disease course, e.g., the amino acid substitution N370S, the GBA genotype proves to poorly predict actual disease presentation in GD patients. Considerable variability in symptoms and general disease severity is documented for several GBA genotypes, even among monozygotic twins (Ferraz et al., 2014). The molecular basis for the interindividual variability in outcome of GCase deficiency among GD patients is not identified yet.

##### Putative advantage of GD heterozygotes

Another intriguing aspect of GD forms the high incidence among Ashkenazim with a disease allele frequency at approximately 0.03–0.04, around 10-fold higher than in non-Jewish populations (Beutler and Grabowski, 2001). The elevated incidence of GD in Ashkenazi Jews is due to four common mutations (Koprivica et al., 2000). The elevated incidence of GD (and other lysosomal storage disorders in glycosphingolipid metabolism such Niemann-Pick disease type B and Tay-Sachs disease) in Ashkenazi populations has led to a great deal of speculation about its cause, ranging from founder effects to a heterozygote advantage. A founder effect as cause seems very unlikely given the small size of the founding Ashkenazi populations in Eastern Europe (Diamond, 1994). The origin of the common N370S mutation in Ashkenazi Jews is thought, based on haplotype data, to have arisen too recently, a mere thousand years ago, to explain the current allele frequency as the result of genetic drift alone (Boas, 2000; Colombo, 2000). The increased allele frequencies of four GBA mutations in Ashkenazi Gaucher patients makes this additionally statistically improbable (Diamond, 1994; Diaz et al., 2000). It therefore has been speculated that GD carriers may be less vulnerable to infectious diseases that cause many victims in city-dwelling populations such as bubonic plaque or tuberculosis. Macrophages are key players in GD and Niemann-Pick disease type B and these cells host *M. tuberculosis*. Evidence for the appealing carrier advantage hypothesis is still missing. Of note, in a zebrafish model of tuberculosis (*M. marianum*) deficiency of several lysosomal hydrolases increases vulnerability for the infection, however, interestingly not that of GCase (Berg et al., 2016; Meijer and Aerts, 2016).

##### Gaucher cells and their secreted markers

Characteristic lipid-laden macrophages accumulate in the spleen, liver, bone marrow, lymph nodes, and lung of GD patients. These Gaucher cells are metabolically active, alternatively activated, macrophages (Boven et al., 2004). GD patients develop low-grade inflammation and coagulation, and show activation of the complement cascade (Hollak et al., 1997; Vissers et al., 2007). Gaucher cells over-express and secrete specific proteins into the circulation of which some are presently employed as biomarkers of body burden of storage macrophages (Ferraz et al., 2014). Examples are chitotriosidase, the human chitinase (Hollak et al., 1994; Bussink et al., 2006), the chemokine CCL18/PARC (Boot et al., 2004) and a soluble fragment of gpNMB (Kramer et al., 2016). Interestingly, increased levels of plasma chitotriosidase also occur with some infectious disease involving macrophages such as Leishmaniasis, tuberculosis, malaria, and leprosy (Hollak et al., 1994; Aerts et al., 2008; Iyer et al., 2009; Di Rosa et al., 2016).

#### Metabolic Adaptations to GCase Deficiency for Better or Worse

Striking metabolic adaptations occur during GCase deficiency (Ferraz et al., 2014). Firstly, increased anabolism of GlcCer to gangliosides takes place, contributing to insulin resistance (Langeveld and Aerts, 2009). Another adaptation involves the cytosol-faced retaining β-glucosidase GBA2. Increased activity of this enzyme during GCase deficiency leads to increased formation of pro-apoptotic Cer from GlcCer. Reducing GBA2 activity, genetically or by means of small compound inhibitors like the iminosugar AMP-DNM, has remarkable beneficial effects in Niemann Pick type C (NPC) mice with a defect in the lysosomal protein NPC1 mediating efflux of cholesterol from lysosomes and secondary GCase deficiency (Nietupski et al., 2012; Marques et al., 2015). AMP-DNM treatment also exerts a neuro-protective effect in mice with Sandhoff disease, another glycosphingolipid storage disorder (Ashe et al., 2011). Of note, GBA2 can act as transglycosylase, transferring the glucose from GlcCer to cholesterol and forming glucosyl-β-cholesterol (GlcChol) in the process (Marques et al., 2016). Another adaptation during GCase is the conversion of accumulating GlcCer in lysosomes by acid ceramidase to its sphingoid base, glucosylsphingosine (GlcSph) (Ferraz et al., 2016). GlcSph reaches the circulation and plasma GlcSph is on average 200-fold elevated in symptomatic type 1 GD patients (Dekker et al., 2011). Measurement of elevated plasma GlcSph has come into use for laboratory confirmation of GD diagnosis (Mirzaian et al., 2015). Albeit clinically distinct diseases, the glycosphingolipidoses show as uniform response to lysosomal GSL accumulation, that is the conversion of storage lipid to its corresponding sphingoid base. In Fabry disease (α-galactosidase deficiency), Krabbe disease (galactocerebrosidase deficiency), GM2 gangliosidosis (β-hexosaminidase deficiency), and Niemann Pick diseases types A and B (acid sphingomyelinase deficiency) the corresponding sphingoid bases of the accumulating substrates (lysoGb3, galactosylsphingosine, lysoGM2, and lysoSM, respectively) are formed and their plasma levels are markedly increased, offering diagnostic possibilities (Ferraz et al., 2014; Mirzaian et al., 2017; Marshall et al., 2019).

#### Pathophysiology

There is compelling evidence for a prominent role of Gaucher cells in GD pathology. Excessive GlcSph stemming from these storage cells is likely pathogenic. It is thought to contribute to the common osteopenia (reduced bone mineral density) in GD patients by impairing osteoblasts (Mistry et al., 2014), to promote α-synuclein aggregation, a hallmark of Parkinson disease (Taguchi et al., 2017), and to underly as auto-antigen in the common gammopathies in GD patients that can evolve into multiple myeloma, a relatively common leukemia in GD patients (Nair et al., 2016). Antigenicity of GlcCer and GlcSph has been postulated to lead to complement cascade activation promoting local tissue inflammation and destruction (Pandey et al., 2017). The diminished cerebral microvascular density in a neuronopathic GD mouse has been attributed to GlcSph based on the observed ability of the sphingoid base to interfere with endothelial cytokinesis *in vitro* (Smith et al., 2018). At present the impact of excessive glucosylated metabolites, like GlcChol, generated by GBA2 activity during GCase deficiency is unknown.

#### Therapies

A very successful therapeutic intervention of type 1 GD is enzyme replacement therapy (ERT), an approach in which patient’s macrophages are supplemented with lacking enzyme by repeated intravenous infusion of therapeutic recombinant GCase (Brady, 2003). To ensure the desired targeting to macrophages, the therapeutic GCase has N-linked glycans with terminal mannose groups to favor uptake by macrophages via mannose-binding lectins like the mannose receptor at the surface of these cells. Two-weekly ERT of type 1 GD patients spectacularly reverses visceral symptoms like hepatosplenomegaly and corrects hematological abnormalities. Unfortunately, ERT does not prevent neurological symptoms due to inability of the enzyme to pass the blood brain barrier. Substrate reduction therapy (SRT) is an alternative registered treatment of type 1 GD. It aims to balance the synthesis of GlcCer with the diminished capacity of GD patients to degrade it (Platt et al., 2001; Aerts et al., 2006). In SRT orally available inhibitors of GCS are employed. Two drugs [Miglustat, *N*-butyl-deoxynojirimycin (NB-DNJ) and Eliglustat (*N*-[(1*R*,2*R*)-1-(2,3-Dihydro-1,4-benzodioxin-6-yl)-1-hydroxy-3-(1-pyrrolidinyl)-2-propanyl] octanamide)] are presently approved for treatment of type 1 GD patients (Figure 4B). Treatment with the more potent and specific Eliglustat is found to result in visceral improvements in patients on a par with ERT (Mistry et al., 2018). Unfortunately, Eliglustat fails to penetrate the brain effectively and can neither be applied to treat neuropathic GD. The design of suitable brain-permeable inhibitors of GCS is investigated and pursued by industry and academic researchers (Shayman and Larsen, 2014). Venglustat (ibiglustat) is developed by Sanofi Genzyme for the treatment of Fabry disease, neuronopathic GD and Parkinson disease. A phase 2 clinical trial (NCT02228460) of Venglustat has recently been conducted to assess short-term safety and effects of the treatment in adult men with Fabry disease. Miglustat is a relatively poor inhibitor of GCs (IC_50_ in the micromolar range) and inhibits off-target intestinal glycosidases and in particular non-lysosomal GBA2 (IC_50_ value in the nanomolar range). Albeit being brain permeable, it is presently not registered as drug to treat neuronopathic GD. Comparable, but superior, iminosugar inhibitors of GCS to Miglustat, like AMP-DNM [*N*-(5-adamantane-1-yl-methoxy-pentyl)-deoxynojirimycin] and its idose-configured analog, were developed some decades ago (Wennekes et al., 2010). These are orally available high nanomolar GCS inhibitors that have impact on GSL metabolism in brain of mice and were found to ameliorate the disease course in mice with NPC disease and Sandhoff disease (Nietupski et al., 2012; Marshall et al., 2019; Figure 4B). Through medicinal chemistry more potent and specific GCS inhibitors have been designed using ido-AMP-DNM as scaffold (Ghisaidoobe et al., 2014). It should be noted that reduction of GlcCer formation by GCS results in the reduction of GlcCer and the metabolically upstream GSLs such as globosides and gangliosides. It therefore has the potential to ameliorate lysosomal storage disorders in which such compounds accumulate, such as GD, Fabry disease, GM2 gangliosidosis, Tay-Sachs disease, Sandhoff disease, GM1 gangliosidosis, and NPC disease.

### Pharmacological Modulation of GSLs: New Avenue for Infection Control?

#### Therapeutic GCS Inhibitors

Given the demonstrated importance of GSLs in infection and control thereof by the immune system (see section “Glycosphingolipids and Infection”) and given the recent application of well tolerated inhibitors of GSL biosynthesis in GD patients (see section “Lysosomal Glycosphingolipid Storage Disorders and Therapy”), it is here proposed to consider use of such compounds to control and/or prevent specific infections. We argue the hypothetical case that glycosphingolipid lowering is feasible and tolerated and might be considered as new therapeutic avenue for specific infectious diseases.

#### Supportive Findings

Experimental support for the use of GSL-lowering agent to combat infection can be found in literature. For example, P-fimbriated *E. coli* are pathogenic in uncomplicated pyelonephritis. The P fimbriae of the bacterium bind the globosides such as Gb3 and Gb4 (Källenius et al., 1980; Leffler and Svanborg-Edén, 1981). When mice were fed with a high dose of the GSL biosynthesis inhibitor NB-DNJ, they showed a decrease in levels of GSLs and less susceptibility for urinary tract infection by P-fimbriated *E. coli* (Svensson et al., 2003).

Along the same line is the outcome of elegant studies by Inokuchi et al. (2015, 2018). Studies with genetically modified mice lacking specific gangliosides (GM3S-null mice expressing o-series gangliosides, but not a- or b-series gangliosides and GM2/GD2S-null mice expressing GM3 and GD3, but no other gangliosides) rendered new insights regarding the importance of the presence of specific gangliosides during allergic and autoimmune diseases. It appears that reduction of specific gangliosides might offer treatment for specific disorders of the immune system (Inokuchi et al., 2015). One example in this direction is allergic asthma, a type 1 hypersensitivity reaction, in which CD4+ T cells mediate Th2 cytokine (IL-4 and IL-13) production, stimulating B cells to produce IgE antibodies. GM3S-null mice show striking reduction of allergic airway responses normally induced by ovalbumin (OVA) inhalation (Nagafuku et al., 2012).

Noteworthy are also the beneficial findings made with GSL lowering agents for systemic lupus erythematosus (SLE). This autoimmune disease manifests with chronic inflammation and leads to damage of tissue (Tsokos, 2011; Kidani and Bensinger, 2014). In SLE there is a prominent T cell dysfunction: CD4+ T cells from patients have lipid rafts with an altered GSL composition. Elevated GSLs (LacCer, Gb3, and GM1) in SLE patients are linked to increased expression of LXRb. The inhibition of GSL biosynthesis with NB-DNJ has been reported to correct CD4+ T cell signaling. In addition, it decreased anti-dsDNA antibody production by autologous B cells in SLE patients (McDonald et al., 2014).

Pharmacological reduction of GSLs is reported to exert beneficial anti-inflammatory effects. GSL-lowering by oral AMP-DNM treatment of mice with trinitrobenzene sulphonic acid (TNBS)- and oxazolone-induced colitis reduced disease severity and edema and suppressed inflammation (Shen et al., 2004). Prominent anti-inflammatory effects of AMP-DNM treatment were also noted for the liver and adipose tissue of obese rodents (Bijl et al., 2009; van Eijk et al., 2009; Lombardo et al., 2012). Non-Alcoholic Fatty Liver Disease (NAFLD) develops during the metabolic syndrome. NALFD involves liver abnormalities ranging from steatosis (fat accumulation) to non-alcoholic steatohepatitis (NASH) including fibrosis and inflammation. Treatment of obese mice with AMP-DNM not only corrects glucose homeostasis and restores insulin signaling in the liver but also reduces inflammation in the tissue (Bijl et al., 2009). A subsequent study revealed that a treatment with the GSL-lowering AMP-DNM is able to significantly correct pre-existing NASH (Lombardo et al., 2012). During obesity, inflammation of adipose tissue is thought to significantly contribute to pathophysiology. AMP-DNM treatment of obese mice improves the status of adipose tissue in many aspects, including a prominent reduction of inflammation (van Eijk et al., 2009). The treatment also leads to decreased iNKT cell activation in adipose tissue of lean mice (Rakhshandehroo et al., 2019).

#### Fungal GlcCer and GCS as Target

Fungal infections (cryptococcosis, candidiasis, aspergillosis, and pneumocystosis) are clinically highly relevant. Shortcomings of current anti-fungal drugs are toxicity and drug resistance. Moreover, not all fungi respond to particular drugs. A recently recognized universal target for combatting fungi is GlcCer (Rollin-Pinheiro et al., 2016; Fernandes et al., 2018). This lipid proves to be crucial for the virulence of pathogenic fungi in plants and man. The latter include *C. albicans, Cryptococcus neoformans*, and *Aspergillus fumigatus*. GlcCer is in particular critical for survival of fungi in neutral and alkaline environments. Indeed, antibodies to fungal GlcCer were found to exert antifungal effects at such conditions. More recently, desired lowering of fungal GlcCer can be reached by reducing the biosynthesis of the lipid. Well tolerated acylhydrazones have been identified as specific inhibitors of fungal GCS, an enzyme that fundamentally differs from the mammalian counterpart and that is not inhibited by acylhydrazones (Lazzarini et al., 2018; Mor et al., 2018). Pharmaceutical reduction of fungal GlcCer is now envisioned as new opportunity to combat fungal infections, including cryptococcosis.

#### Neuraminidase Inhibitors as Anti-influenza Viral Agents

In the 1990’s inhibitors of neuraminidase have been designed for prophylaxis and treatment of influenza. The surface envelope of the influenza virus contains the glycoproteins hemagglutinin and neuraminidase. Hemagglutinin mediates viral attachment to the cell surface receptor containing a terminal N-acetylneuraminic acid residue attached α-(King, 1956; Merrill, 2011) or α-(King, 1956; Gault et al., 2010) to a galactose. By a variety of techniques, like thin-layer chromatography overlay assays and mass spectrometry, the nature of lipid receptors has been identified (Meisen et al., 2012; Hidari et al., 2013). The viral neuraminidase is essential for timely release of the virus from the cellular anchor. The neuraminidase inhibitors zanamivir, laninamivir, oseltamivir, and peramivir have been shown to be effective against most influenza strains, but resistance to specific drugs has developed in some cases (Dobson et al., 2015; Laborda et al., 2016). Some of the neuraminidase inhibitors are also employed as useful research tools in investigations on ganglioside biology (Crain and Shen, 2004; Moore et al., 2007). Total internal reflection fluorescence microscopy has been recently successfully employed to investigate the interaction of viruses with ganglioside containing lipid bilayers, the importance of hemagglutinin and neuraminidase in the process and the inhibitory action of zanamivir (Müller et al., 2019).

## Future Lipidomics Challenges

The investigation of lipids has been historically complicated by their intrinsic hydrophobic features and heterogeneous nature. The recent progress in quantitative mass spectrometric detection of lipids has, however, opened a new world for lipidomics. This field is rapid advancing (see Han and Gross, 2003 for an excellent review on the topic). In particular ESI (electrospray ionization) and MALDI (matrix assisted laser desorption/ionization) mass spectrometry methods are nowadays successfully applied in lipidomics (Wang et al., 2019). Besides targeted measurement of specific lipids with MRM (multiple reaction monitoring), non-targeted approaches like shotgun and multi-dimensional lipidomics are increasingly employed (for a recent review on the topic see Bilgin et al., 2016). Improvements have been made in lipid extraction methods (Cruz et al., 2016; Löfgren et al., 2016) and internal standards, such as isotope encoded analogs, become increasingly available (Wisse et al., 2015; Mirzaian et al., 2016; Wang et al., 2017). Derivatization or deacylation of specific lipids may assist their quantitative detection (Mirzaian et al., 2017; Ma et al., 2019). An important new development is the availability of techniques to study the biology of lipids in living cells. Fluorescent NBD and BODIPY tagged lipids have been used in first generation cell biological investigations and in recent times advances have been made in the generation of photoactivatable, caged, photo-switchable, and tri-functional lipid derivatives assisting the imaging of lipids (reviewed in Laguerre and Schultz, 2018). The spatio-temporal detection of endogenous lipids in cells and tissues still remains a major challenge. QQImaging mass spectrometry (IMS) aims to visualize the location and distribution of metabolites in intact biological samples (see Parrot et al., 2018 for a recent review). One of the ISM techniques employs desorption electrospray ionization (DESI) (Parrot et al., 2018). Minimally destructive DESI-IMS chemical screening is achieved at the μm-scale resolution. Alternatively, MALDI-MS imaging is used to detect locally lipids, including GSLs (Vens-Cappell et al., 2016; Jones et al., 2017; Caughlin et al., 2018; Hunter et al., 2018; Sugiyama and Setou, 2018; Tobias et al., 2018; Enomoto et al., 2019). A new development forms the technology for *in situ* visualization of enzymes involved in glycosphingolipid metabolism. Designed have been fluorescent activity-based probes that covalently label – and visualize – active enzyme molecules through covalent linkage to catalytic nucleophile residues. An example in this direction is the enzyme glucocerebrosidase for which probes have been developed allowing *in situ* monitoring of active enzyme molecules (Witte et al., 2010; Kallemeijn et al., 2012; van Meel et al., 2019).

## Perspectives

Clinical and laboratory research over many decades has revealed that various pathogens require GSLs of host cells for infection. Thus, the modulation of such lipids in host cells could *a priori* be considered as treatment for infection control. An obvious provision for such approach is that it does no harm. Any significant reduction of GSLs has been considered for a long time to yield considerable side-effects, likely translating in severe symptoms. The long-term outcome of treatment of patients suffering from GD with agents that reduce GSLs is, however, remarkably positive. No major side-effects are observed in individuals treated for a number of years (Mistry et al., 2018; Lewis and Gaemers, 2019). So far, the agents used do not achieve significant reduction in GSLs in the brain, however, a new generation of compounds aiming at that is being tested at the moment. The near future will learn whether it is feasible to safely reduce GSLs in cells and tissues, including the brain. Next it will have to be established whether such reductions are indeed effective for infection control.

Enormous progress has been made in knowledge on the role of GSLs in various kinds of infection and the immune system’s response to this. At this moment much of the knowledge is still descriptive and little translation to preventing and/or treating infections has been accomplished. Genetics and genomics may not provide answers to all questions. It remains essential to acquire fundamental insight on metabolism of GSLs in these gene-oriented times. Such insight will essentially contribute to the design/development of suitable agents than can subtly modulate GSLs as desired for infection control.

This review focusses on pharmacological ways to reduce GSL levels. A fundamentally different approach to target GSL-pathogen interactions that has also been conceived is the design of potent carbohydrate-type competitors of bacterial adhesion (Schengrund, 2003; Pieters, 2011). Such approach copies more or less the presumed protective effects of oligosaccharides in milk during the colonization of the intestine.

In conclusion, the coming years should reveal whether GSLs may act as valuable target in infection control.

## Author Contributions

All authors contributed to writing the review and preparing figures.

## Conflict of Interest

The authors declare that the research was conducted in the absence of any commercial or financial relationships that could be construed as a potential conflict of interest.
